# Dysphagia Weakly Correlates With Other Patient-Reported Outcomes After Anterior Cervical Discectomy and Fusion

**DOI:** 10.7759/cureus.20742

**Published:** 2021-12-27

**Authors:** Philip Zakko, Rafid Kasir, Nai-Wei Chen, Daniel Park

**Affiliations:** 1 Department of Orthopaedic Surgery, Beaumont Health, Royal Oak, USA

**Keywords:** anterior cervical discectomy and fusion, patient-reported outcomes, bazaz, eat-10, dysphagia

## Abstract

Introduction

Dysphagia is a common complication after anterior cervical discectomy and fusion (ACDF), but it is not a routinely asked question in legacy patient-reported outcome measures (PROMs). This study analyzes whether there are associations between dysphagia and legacy outcome measures.

Methods

We retrospectively reviewed 168 patients who underwent ACDF surgery from 2017 to 2019 at a single institution. Demographics, anthropometric data, Neck Disability Index (NDI), Visual Analog Scale (VAS)-Arm and VAS-Neck Pain scores, Patient-Reported Outcomes Measurement Information System (PROMIS)-Physical and PROMIS-Mental scores, Charlson Comorbidity Index (CCI), and Eating Assessment Tool-10 (EAT-10) were obtained for each patient preoperatively and at one, three, six, and 12 months postoperatively. Pearson’s correlation coefficients were used to evaluate the bivariate correlations between legacy, PROMIS, and EAT-10 measures.

Results

Significant but weak correlations existed between NDI and EAT-10 at one, three, and six months postoperatively (correlation coefficient (R) = 0.31, 0.42, and 0.34 at one, three, and six months, p < 0.001) and VAS-Neck Pain and EAT-10 scores at one, three, and six months postoperatively (R = 0.27, 0.30, and 0.28 at one, three, and six months, p ≤ 0.004). Both PROMIS-Physical and PROMIS-Mental scores showed significant but weak correlations with EAT-10 scores at three and six months postoperatively (R = -0.29 and -0.25, p ≤ 0.01, at three months and R = -0.25 and -0.28, p < 0.01, at six months). In all comparisons of EAT-10 scores with legacy outcome measures, the significance of correlations disappeared by 12 months postoperatively. In addition, there was a positive association between CCI and EAT-10 score (β = 0.37, p < 0.05).

Conclusion

Weak correlations exist between self-reported dysphagia scores and legacy patient-reported outcome measures in patients undergoing ACDF. The correlation strength decreases over time; therefore, dysphagia scores should be reported separately when looking at outcomes after ACDF. Patients with more comorbidities are also at increased risk for dysphagia.

## Introduction

Anterior cervical discectomy and fusion (ACDF) is one of the most commonly performed spinal surgeries in the United States [1]. Patient postoperative outcomes are generally favorable with patient self-reported success ranging from 85% to 95% [2]; however, ACDF is not without complications. Morbidity rates for ACDF vary from 13.2% to 19.3%. Complications include postoperative hematoma, epidural hematoma, Horner’s syndrome, exacerbation of myelopathy, pseudoarthrosis, and adjacent segment disease. Dysphagia, however, is the most common complication with rates reported from 1.7% to 67% [1-4]. Nevertheless, dysphagia is not routinely reported when assessing outcomes after ACDF [2,5-8].

Patient-reported outcomes (PROs) after surgery are essential metrics in determining the success of any operation. A recent study discussed how PROs, including legacy and Patient-Reported Outcomes Measurement Information System (PROMIS), have revolutionized the definition of success in cervical spine surgery [8]. Legacy measures include the Neck Disability Index (NDI), Visual Analog Scale (VAS)-Arm and VAS-Neck Pain scores, and generic health questionnaires such as EuroQol-5D (EQ-5D) and 36-Item Short Form Survey (SF-36). Separate from legacy measures, PROMIS is a newer validated system of PRO measurements that was developed to improve the reporting and quantification of changes in PROs. PROMIS consists of 102 adult domains with a total of 1903 items or questions. Of these 102 domains, those relating to physical function and mental health are most commonly utilized in the spine literature [9]. While both legacy and PROMIS-Physical/PROMIS-Mental scores are excellent tools in capturing PROs after surgery, neither includes a question that specifically assesses the most common complication after ACDF - a patient’s ability to swallow.

Various measures have been utilized to assess patients’ self-reported dysphagia after ACDF. The most commonly utilized scales include the Bazaz and SWAL-QoL, both of which have limitations in validity and reliability [3,10-12]. The Eating Assessment Tool-10 (EAT-10) is a symptom-specific 10-item clinical instrument that was developed to assess patient self-reported dysphagia symptom severity [10]. Compared with other metrics including the Bazaz, the EAT-10 was found to be the most accurate tool in assessing postoperative dysphagia [13].

The primary objective of this study is to quantitatively assess whether legacy and PROMIS-Physical/PROMIS-Mental measures correlate with self-reported dysphagia as assessed by EAT-10. Since dysphagia is the most common complication after ACDF, if correlations are not found, it may be necessary to collect dysphagia data on all ACDF patients to fully assess their PROs after any cervical surgery. In addition, we also assess the effects of baseline patient characteristics on dysphagia (EAT-10) after ACDF using the regression approach.

## Materials and methods

Study design and selection of participants

This retrospective study was approved by the Institutional Review Board of Beaumont Research Institute. All patients who underwent ACDF between January 2017 and December 2019 by the senior author (DP) at a single institution were identified in electronic medical records and the operating surgeon’s surgical database. All operative and nonoperative patients seen in the senior author’s office are required to answer various patient-reported outcome measures (PROMs) on a provided iPad prior to their office appointment.

Of those patients who underwent ACDF between January 2017 and December 2019, patients were included if they are older than age 18 years, if they underwent a single or multilevel ACDF for primary or revision purposes, and if follow-up occurred in the senior author’s private orthopedic clinic. Patients were excluded if they were not cognitively able to complete questionnaires at baseline; were seen in the emergency department prior to surgery, thus having no preoperative PROs; or if postoperative follow-up was performed at the tertiary care resident-run orthopedic clinic, thus having no postoperative PROs collected. A total of 168 patients met the selection criteria and were included in the study.

Surgical technique

An anterior approach to the cervical spine was utilized for all patients. A left-sided transverse skin incision was made as is preferred by the senior author. After dissection through the platysma, the soft tissues were undermined, and a standard Smith-Robinson approach was utilized. Self-retaining retractors were used, and the endotracheal cuff was routinely deflated and reinflated. Dexamethasone 8-10 mg IV was administered commonly by the anesthesia team, and no local steroids in the wound were utilized. Variable plates (Atlantis plate, Medtronic, Minneapolis, MN, USA) were used, and PEEK interbody cages (Stryker Corporation, Kalamazoo, MI, USA) were implanted in all patients.

Study variables

Baseline patient characteristics, including age, sex, body mass index (BMI), Charlson Comorbidity Index (CCI), daily opioid use (yes/no), and depression (yes/no), were collected. Neck Disability Index (NDI), Visual Analog Scale (VAS) pain scores, PROMIS-Physical and PROMIS-Mental scores, and Eating Assessment Tool-10 (EAT-10) were obtained for each patient preoperatively and at one, three, six, and 12 months postoperatively.

Statistical analysis

Descriptive analyses were used to summarize the baseline characteristics of patients. Continuous and categorical variables were expressed as means (standard deviations (SDs)) and frequencies (percentages), respectively. Bivariate correlations were used to examine legacy and PROMIS measures with self-reported dysphagia (EAT-10) at each preoperative and postoperative time point. To increase the stabilization of estimate of correlation coefficients and reduce the odds of errors of inference resulting from pairwise deletion or available case analysis on missing follow-up observations in calculating the Pearson’s correlation coefficients, missing observations were imputed by the procedure of multiple imputation through the fully conditional specification method, accounting for the arbitrary missing patterns [14]. Multivariable linear mixed-effects regression, accounting for repeated measures, was used to assess the association between patient characteristics and EAT-10 scores. To further characterize the variation in the severity of self-reported dysphagia (EAT-10 score: <3, 3-15, and >15) by obesity status (BMI ≥ 30 versus BMI < 30) and comorbid conditions (CCI ≥ 1 versus CCI < 1), respectively, across preoperative and postoperative time points, the ordinal mixed-effects logistic regression was employed to estimate the corresponding percentages. Cutoffs for EAT-10 were selected as recent literature has found that EAT-10 ≥ 3 is indicative of oropharyngeal dysphagia [10] and EAT-10 > 15 is indicative of aspiration risk [12]. The analyzed results were combined from 100 imputed datasets, accounting for the additional variability introduced by the multiple imputation [15]. All tests were two-sided with a p-value < 0.05 considered to be statistically significant. Analyses were performed using SAS version 9.4 (SAS Institute Inc., Cary, NC, USA).

## Results

**Figure 1:**
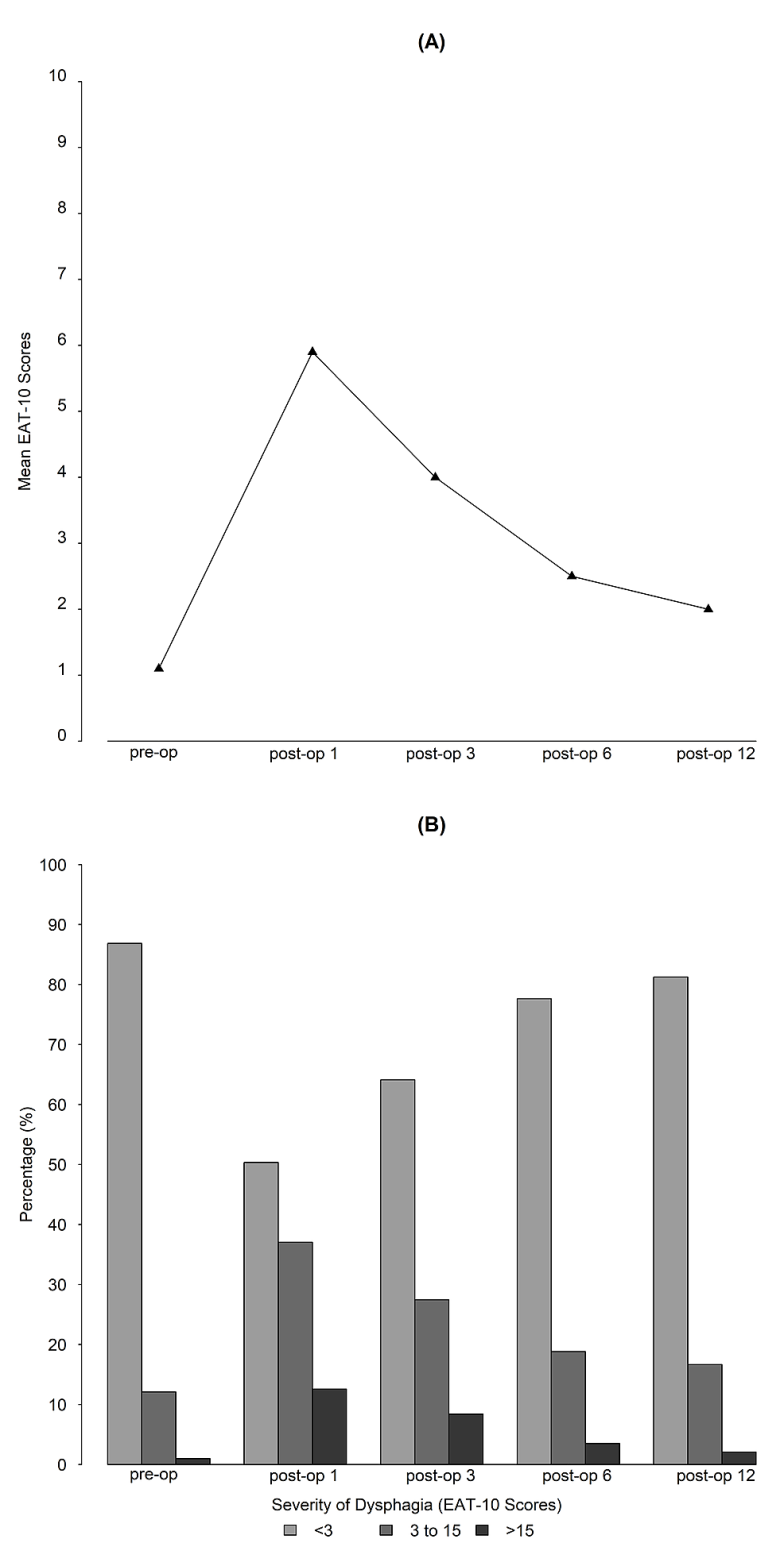
The observed raw percentage of patients with self-reported dysphagia and the mean dysphagia score at each time point

Of 254 patients who underwent ACDF from 2017 to 2019, 168 patients met the selection criteria and were included in this study. The average age of patients was 57 (SD: 12), and 52.4% of the patients were females. Baseline patient characteristics are summarized in Table 1. The observed raw percentage of patients with self-reported dysphagia and the mean dysphagia score at each time point are presented in Figure 1.

Bivariate correlations, NDI versus EAT-10, PROMIS versus EAT-10, and VAS-Neck Pain versus EAT-10 scores are illustrated in Table 2. The results indicate that positive weak correlations existed between NDI and EAT-10 at one, three, and six months postoperatively (correlation coefficient (R): 0.31, 0.42, and 0.34 at one, three, and six months, p < 0.001). There were positive weak correlations between VAS-Neck Pain and EAT-10 scores at one, three, and six months postoperatively (R = 0.27, 0.30, and 0.28 at one, three, and six months, p ≤ 0.004). Both PROMIS-Physical and PROMIS-Mental scores showed weak negative correlations with EAT-10 scores at three and six months postoperatively (R = -0.29 and -0.25, p ≤ 0.01, at three months and R = -0.25 and -0.28, p < 0.01, at six months). Additionally, the correlations became weaker as time after surgery passed and lost significance by 12 months postoperatively.

Table 3 shows that CCI was positively associated with EAT-10 scores (β = 0.37, p = 0.048), whereas smoking, depression, and BMI were not significantly associated with EAT-10 scores. We further categorized EAT-10 scores in terms of severity (no dysphagia, <3; mild dysphagia, 3-15; and severe dysphagia, >15). There was a trend for patients with a BMI ≥ 30 and CCI ≥ 1 to have mild and severe self-reported dysphagia compared with their counterparts, especially at three, six, and 12 months postoperatively (Table 4).

Regarding surgical and postoperative complications, there were no cases of epidural hematoma, symptomatic recurrent laryngeal nerve palsy, cerebrospinal fluid leak, wound infection, esophageal perforation, or Horner’s syndrome. Early complications included one case each of pulmonary embolism, respiratory failure requiring reintubation due to hematoma, urinary retention, C5 nerve palsy, and symptomatic hematoma from a postoperative fall that resolved with monitoring. Late complications included one case each of odynophagia and hardware screw failure requiring revision.

**Table 1:** Demographic and clinical characteristics of the study patients (n = 168) Abbreviations: BMI, body mass index. ^§^ For continuous variables, means ± standard deviations were presented. For categorical variables, frequencies and percentages were presented.

Variables^§^	
Age	57.4 ± 12.1
Gender	
Female	88 (52.4%)
Male	80 (47.6%)
BMI	30.0 ± 5.6
Charlson Comorbidity Index	1.1 ± 2.3
Opioid use	
Yes	46 (27.4%)
No	122 (72.6%)
Depression	
Yes	46 (27.4%)
No	122 (72.6%)
Anxiety	
Yes	44 (26.2%)
No	124 (73.8%)
Smoke	
Yes	23 (13.7%)
No	145 (86.3%)
Alcohol use	
Yes	9 (5.4%)
No	159 (94.6%)

**Table 2:** Correlation between legacy/PROMIS measures and EAT-10 scores Abbreviations: PROMIS, Patient-Reported Outcomes Measurement Information System; NDI, Neck Disability Index; VAS, Visual Analog Scale; p, p-value. ^§^ Pearson’s correlation coefficients were assessed at each observed time point, and the results were pooled from 100 imputed datasets.

Legacy/PROMIS measure^§^	Preoperative	Postoperative time (month)			
		1	3	6	12
NDI	0.04 (p = 0.615)	0.31 (p < 0.001)	0.42 (p < 0.001)	0.34 (p < 0.001)	0.25 (p = 0.012)
VAS-Neck Pain	0.09 (p = 0.292)	0.27 (p = 0.001)	0.30 (p < 0.001)	0.28 (p = 0.004)	0.11 (p = 0.295)
PROMIS-Physical	-0.05 (p = 0.627)	-0.12 (p = 0.149)	-0.29 (p = 0.001)	-0.25 (p = 0.010)	-0.17 (p = 0.111)
PROMIS-Mental	-0.11 (p = 0.220)	-0.10 (p = 0.240)	-0.25 (p = 0.006)	-0.28 (p = 0.003)	-0.15 (p = 0.131)

**Table 3:** Effects of patient characteristics on EAT-10 scores Abbreviations: BMI, body mass index; CCI, Charlson Comorbidity Index.

Variables	Linear mixed regression		
	β	(95% CI)	p-value
Age	0.01	(-0.05 to 0.08)	0.711
Sex (female versus male)	0.40	(-1.16 to 1.96)	0.599
BMI	-0.10	(-0.23 to 0.03)	0.122
CCI	0.37	(0.004 to 0.74)	0.048
Opioid use (yes versus no)	1.23	(-0.40 to 2.87)	0.132
Depression (yes versus no)	1.40	(-0.31 to 3.11)	0.103
Smoke (yes versus no)	-0.77	(-2.98 to 1.44)	0.479
Alcohol use (yes versus no)	-1.97	(-5.64 to 1.70)	0.276
Time (reference: preoperative)			
Postoperative – one month	4.99	(3.80 to 6.17)	<0.001
Postoperative – three months	3.12	(1.95 to 4.30)	<0.001
Postoperative – six months	1.78	(0.53 to 3.03)	0.008
Postoperative – 12 months	1.38	(-0.02 to 2.78)	0.053

**Table 4:** Estimated percentages on the severity of dysphagia by obesity status and comorbidity conditions across preoperative and postoperative time points Abbreviations: BMI, body mass index; CCI, Charlson Comorbidity Index. ^§^ Estimated percentages on the severity of dysphagia were calculated using the ordinal mixed-effects logistic regression. For the obesity status, obesity status (BMI ≥ 30 versus BMI < 30), time, and interaction between obesity status and time were used in a regression model. For comorbidity conditions, comorbidity conditions (CCI ≥ 1 versus CCI < 1), time, and interaction between comorbidity conditions and time were used in a regression model.

EAT-10 scores	Preoperative		Postoperative time (month)							
			1		3		6		12	
Severity^§^	BMI < 30	BMI ≥ 30	BMI < 30	BMI ≥ 30	BMI < 30	BMI ≥ 30	BMI < 30	BMI ≥ 30	BMI < 30	BMI ≥ 30
<3	95.2%	91.2%	64.9%	47.1%	75.5%	60.9%	84.7%	74.2%	89.7%	82.1%
3–15	4.6%	8.4%	32.8%	48.7%	23.1%	36.5%	14.5%	24.3%	9.8%	17%
>15	0.2%	0.4%	2.3%	4.2%	1.4%	2.6%	0.8%	1.5%	0.5%	0.9%
Severity^§^	CCI < 1	CCI ≥ 1	CCI < 1	CCI ≥ 1	CCI < 1	CCI ≥ 1	CCI < 1	CCI ≥ 1	CCI < 1	CCI ≥ 1
<3	94.8%	90.6%	39.6%	44.2%	69.4%	48.5%	82.8%	70.5%	86.6%	74.7%
3–15	4.9%	9.0%	55.1%	51.2%	28.8%	47.5%	16.3%	27.7%	12.7%	23.9%
>15	0.2%	0.4%	5.3%	4.6%	1.8%	4%	0.9%	1.8%	0.7%	1.4%

## Discussion

Patient-reported outcome measures (PROMs) are utilized to help determine success after surgical procedures. In cervical spine surgery, legacy (NDI, VAS-Neck/VAS-Arm, etc.) and PROMIS-Physical/PROMIS-Mental scores are routinely collected for this purpose; however, these scores do not specifically ask about the most common complication after ACDF surgery - dysphagia. Historically, dysphagia was not as widely discussed when initial studies on cervical spine outcome measures were performed, possibly explaining the lack of cervical spine literature where self-reported dysphagia was an outcome. The primary objective of our study was to see if correlations exist between self-reported dysphagia and PROMs. While weak correlations exist between EAT-10 and NDI, VAS-Neck, and PROMIS-Physical/PROMIS-Mental scores up to six months postoperatively, by 12 months postoperatively, these weak correlations became weaker and lost significance. Our secondary objective was to assess patient-specific risk factors for self-reported dysphagia after ACDF. We found that CCI was positively associated with EAT-10 scores and that patients with a BMI ≥ 30 and a CCI ≥ 1 trended toward having mild or severe dysphagia compared with their healthier counterparts. These findings suggest that EAT-10 scores should be collected on all patients to fully assess their PROMs after ACDF.

The current global spine literature is not lacking in studies evaluating outcomes after ACDF surgery. However, the most common complication after ACDF, dysphagia, is not always included as an outcome measure. A retrospective study on data from 2011 to 2016 analyzed patient-reported outcomes after ACDF in 318 Danish patients. With a one-year minimal follow-up period, the study included all legacy outcome measures but did not include any measures on dysphagia [16]. Another study by Patel et al. looked at the association between patient activation and PROs after ACDF. They collected NDI, SF-12, and VAS pain scores but did not look at dysphagia [17]. Buttermann prospectively evaluated PROs over 10 years following ACDF; dysphagia was not included as an outcome measure [2]. Given that dysphagia is the most common complication after ACDF [1,3,4,18], one could argue that dysphagia should be included in any assessment of PROs after ACDF.

Our discovery that self-reported dysphagia has weak initial correlations with NDI, VAS-Neck, and PROMIS-Physical/PROMIS-Mental scores, which become even weaker and lose significance over time, further suggests that self-reported dysphagia should be collected as an outcome measure in all ACDF studies. It is important to realize that legacy and PROMIS-Physical/PROMIS-Mental outcome measures do not ask direct questions regarding dysphagia.

The NDI is made up of 10 scored sections including neck pain intensity, personal care (not relating to dysphagia), lifting objects, reading, headaches, concentration, working, driving, sleeping, and recreation. No section has an option that relates to dysphagia. However, people may associate dysphagia with difficulty performing personal care such as feeding oneself. The ability to engage in recreational activities may also be affected by dysphagia. For instance, patients may struggle to go out to dinner for social events as a result of their dysphagia. Furthermore, VAS-Neck Pain scores utilize a 10-cm visual analog scale that patients mark from no pain to worst pain imaginable - dysphagia is not represented. However, correlations between dysphagia and neck pain may exist if patients associate their dysphagia as a contributing factor to their neck pain.

Finally, PROMIS is a scoring system developed by the NIH that was developed as a way to simplify and standardize the collection of PROs by researchers. PROMIS is divided into multiple domains, including physical function and mental health domains, which are commonly used as orthopedic surgery outcome measures [9]. However, physical function asks questions related to the ability to perform tasks and mobility, while mental health has items related to depression and anxiety. Neither PROMIS-Physical nor PROMIS-Mental domains include questions regarding postoperative dysphagia. However, PROMIS asks about quality of life (QoL), which may be altered if patients are experiencing dysphagia. In addition, when asked how they rate satisfaction with social activities and relationships, patients may respond lower if they have dysphagia as they may struggle to attend social events such as going to restaurants with family and friends. Lastly, patients are asked to rate their fatigue on average, and dysphagia may directly contribute to their fatigue due to decreased oral intake and frustration with their decreased ability to swallow.

These weak correlations also suggest that self-reported dysphagia does in fact cause patient morbidity and QoL alterations. Following ACDF, dysphagia typically plateaus after six months [19]; thus, the effects of dysphagia on morbidity and QoL likely disappear at long-term follow-up. However, we believe that the legacy and PROMIS-Physical/PROMIS-Mental measures are not sensitive enough to decipher the magnitude of these effects. In addition, about 2% of patients in our study suffered from severe self-reported dysphagia at 12-month follow-up. While mild in many patients, some patients suffer from moderate to severe dysphagia lasting longer than six months and need to be reported as such [20].

We also aimed to assess patient-specific risk factors for self-reported dysphagia after ACDF and found a positive association between CCI and EAT-10 scores and that patients with a BMI ≥ 30 and a CCI ≥ 1 trended toward having mild or severe dysphagia compared with their healthier counterparts. Other published studies have looked at risk factors for dysphagia after ACDF. From 2013 to 2017, Yew et al. looked at risk factors for postoperative dysphagia using the EAT-10. They found that obstructive sleep apnea, asthma, and increased American Society of Anesthesiologists (ASA) score were associated with increased risk of dysphagia in the later postoperative period [3]. Another study published in 2019 by Vaishnav et al. looked at single-level ACDF risk factors for dysphagia in the early six- and 12-week postoperative time points using the Swallowing Quality of Life (SWAL-QoL) dysphagia score. Preoperative SWAL-QoL scores and operative time were found to be predictors of dysphagia [21]. This study had the disadvantage of using the SWAL-QoL over EAT-10 given dysphagia scores such as Bazaz and SWAL-QoL exhibit less reliability and validity compared with EAT-10 [3,10-12]. A recent study by Ohba et al. used the EAT-10 and the Hyodo-Komagane score with endoscopic evaluation to assess risk factors for postoperative dysphagia after ACDF. This study found that aging and smoking were risk factors for transient dysphagia [22]. Other studies have found risk factors for dysphagia after ACDF to include aging, operation time, blood loss, smoking, endocrine disorders, and an increased number of upper cervical spine surgeries [23-25]. Dysphagia is an important complication to record as hospitalized patients with dysphagia have poorer outcomes and require higher economic costs than those without [26]. The great variance within the literature and our current study suggest that a multitude of factors predispose patients to dysphagia after ACDF.

To our best knowledge, this is the first study to compare self-reported dysphagia scores to legacy and PROMIS outcome measures. The patients in this study exhibited clinically significant improvements in VAS-Neck Pain scores and NDI scores, which meet standards in the literature for minimal clinically important differences (MCIDs) [27,28]. Our observed self-reported dysphagia rates of 22.4% at six months postoperatively and 18.9% at 12 months postoperatively were also similar to those seen in other studies [1,29].

The current study is not without limitations. This study was a retrospective review without patient randomization and with potential confounders. In addition, while EAT-10 is currently the most accurate and reliable noninvasive outcome measure to assess dysphagia, it is a subjective score calculated from a patient’s report. To better assess dysphagia, more invasive techniques such as endoscopic and videofluoroscopic swallow studies could be performed to assess objectively swallowing dysfunction. Future studies may benefit from these techniques in combination with EAT-10.

## Conclusions

This study found that weak correlations exist between self-reported dysphagia scores and legacy patient-reported outcome measures in patients undergoing ACDF. By 12 months postoperatively, these weak correlations lost significance. In addition, patients with more comorbidities prior to surgery were more likely to have dysphagia after surgery. Obese patients or those with a CCI ≥ 1 trended toward having mild or severe dysphagia compared with their healthier counterparts. Given the lack of correlation between dysphagia and other patient-reported outcome measures, self-reported dysphagia scores should be reported separately when looking at outcomes after ACDF.
